# Variations and Opportunities in Postnatal Management of Hemolytic Disease of the Fetus and Newborn

**DOI:** 10.1001/jamanetworkopen.2024.54330

**Published:** 2025-01-10

**Authors:** Derek P. de Winter, E. J. T. (Joanne) Verweij, Anne Debeer, Roland Devlieger, Liesbeth Lewi, Sarah Verbeeck, Paul Maurice, Jean-Marie Jouannic, Marie-Gabrielle Guillemin, Agnès Mailloux, Maria Cristina Pessoa dos Santos, Cynthia Amaral de Moura Sá Pacheco, Maria Elisabeth Lopes Moreira, Marcella Martins de Vasconcelos Vaena, Kajsa Bohlin, Eleonor Tiblad, Emilie Thorup, Olav Bjørn Petersen, Maria Sanchez-Holgado, Aurora Viejo Llorente, Borna Poljak, Asma Khalil, Kévin Le Duc, Louise Ghesquiere, Jana Lozar Krivec, Aneta Soltirovska-Šalamon, Christof Dame, Jessica D. Blank, Alexander Hohnecker, Matthew Saxonhouse, Ngina K. Connors, Annegret Geipel, Johanna Rath, Smriti Prasad, Lizelle van Wyk, Lut Geerts, Rahel Schuler, Nina Thon, Leah Leibovitch, Stav Cohen, Arturo Alejandro Canul-Euan, Edmond Kelly, Kamini Raghuram, Francesco Cavigioli, Sofia Fatima Guiseppina Colombo, Ziju Elanjikal, Jessica Brayley, Daniel Pfurtscheller, Gerhard Pichler, Ángel Guillermo Alcázar Grisi, Edgar Juan José Chávez Navarro, Joana Pereira-Nunes, Henrique Soares, Ming Zhou, María José Garcia Borau, Elisenda Moliner Calderón, Maria Fernanda Galletti, Gonzalo Luis Mariani, David Mackin, Fergal Malone, Andrea Lampland, Wing Ting Tse, James Castleman, Johanna G. van der Bom, Masja de Haas, Enrico Lopriore

**Affiliations:** 1 Division of Neonatology, Department of Pediatrics, Willem-Alexander Children’s Hospital, Leiden University Medical Center, Leiden, the Netherlands; 2 Division of Fetal Medicine, Department of Obstetrics, Leiden University Medical Center, Leiden, the Netherlands; 3 Department of Immunohematology Diagnostic Services, Sanquin Diagnostic Services, Amsterdam, the Netherlands; 4 Department of Development and Regeneration, KU Leuven, Leuven, Belgium; 5 Department of Neonatology, University Hospitals Leuven, Leuven, Belgium; 6 Department of Obstetrics and Gynaecology, University Hospitals Leuven, Leuven, Belgium; 7 French National Referral Center in Perinatal Hemobiology and Fetal Medicine Department, Trousseau Hospital, AP-HP, Sorbonne University, Paris, France; 8 French National Referral Center in Perinatal Hemobiology, Saint-Antoine Hospital, AP-HP, Sorbonne University, Paris, France; 9 Instituto Nacional de Saúde da Mulher, da Criança e do Adolescente Fernandes Figueira (IFF/Fiocruz), Rio de Janeiro, Brazil; 10 Department of Neonatology, Karolinska University Hospital and Karolinska Institutet, Stockholm, Sweden; 11 Division of Clinical Epidemiology, Department of Medicine, Karolinska Institutet, Stockholm, Sweden; 12 Department of Obstetrics and Gynecology, Umeå University Hospital, Umeå, Sweden; 13 Department of Gynecology, Fertility and Obstetrics, Copenhagen University Hospital, Rigshospitalet, Copenhagen, Denmark; 14 Department of Clinical Medicine, University of Copenhagen, Copenhagen, Denmark; 15 Department of Neonatology, La Paz University Hospital, Madrid, Spain; 16 Department of Hematology and Hemotherapy, La Paz University Hospital, Madrid, Spain; 17 Fetal Medicine Unit, Liverpool Women’s Hospital NHS Foundation Trust, Liverpool, United Kingdom; 18 Fetal Medicine Unit, St George’s Hospital, St George’s University of London, London, United Kingdom; 19 Department of Neonatology, Université de Lille, CHU Lille, Lille, France; 20 Department of Obstetrics, Université de Lille, CHU Lille, Lille, France; 21 Department of Neonatology, University Children’s Hospital, University Medical Centre Ljubljana, Ljubljana, Slovenia; 22 Faculty of Medicine, University of Ljubljana, Ljubljana, Slovenia; 23 Department of Neonatology, Charité-Universitätsmedizin Berlin, Berlin, Germany; 24 Kinderklinik des Klinikums Dritter Orden, Klinikums Dritter Orden, München, Germany; 25 Wake Forest School of Medicine, Charlotte, North Carolina; 26 Atrium Healthcare Levine Children’s Hospital, Charlotte, North Carolina; 27 Department of Obstetrics and Gynecology, Atrium Healthcare Carolinas Medical Center, Charlotte, North Carolina; 28 Department of Obstetrics and Prenatal Medicine, University Hospital Bonn, Bonn, Germany; 29 Department of Paediatrics and Child Health, Faculty of Medicine and Health Sciences, Stellenbosch University and Tygerberg Hospital, Cape Town, South Africa; 30 Tygerberg Academic hospital, Faculty of Medicine and Health Sciences, Department of Obstetrics and Gynaecology, Stellenbosch University, Cape Town, South Africa; 31 Department of General Pediatrics and Neonatology, Justus-Liebig-University, Giessen, Germany; 32 Department of Neonatology, Sheba Medical Center, Tel Hashomer, Israel; 33 Department of Obstetrics and Gynecology, Sheba Medical Center, Tel-Aviv University, Tel Aviv, Israel; 34 Department of Reproductive and Perinatal Health Research, National Institute of Perinatology, Mexico City, Mexico; 35 Neonatology Division, Department of Pediatrics, Ontario Fetal Centre, Mount Sinai Hospital, University of Toronto, Toronto, Ontario, Canada; 36 Neonatal Intensive Care Unit, Buzzi Children’s Hospital, University of Milan, Milan, Italy; 37 Department of Neonatology, University Hospitals Bristol and Weston NHS Trust, Bristol en Weston-super-Mare, United Kingdom; 38 Division of Neonatology, Department of Paediatrics and Adolescent Medicine, Medical University of Graz, Graz, Austria; 39 Department of Gynecology and Obstetrics, Hospital de La Mujer, La Paz, Bolivia; 40 Department of Pediatrics, Hospital de La Mujer, La Paz, Bolivia; 41 Department of Neonatology, Unidade Local de Saúde de São João, Porto, Portugal; 42 Department of Gynecology-Obstetrics and Pediatrics, Faculty of Medicine, Porto University, Porto, Portugal; 43 Department of Neonatology, Shanghai First Maternity and Infant Hospital, Tongji University School of Medicine, Shanghai, China; 44 Department of Neonatology, Hospital de la Santa Creu i Sant Pau, Barcelona, Spain; 45 Neonatology Division, Hospital Italiano de Buenos Aires/Instituto Universitario Hospital Italiano de Buenos Aires, Buenos Aires, Argentina; 46 Royal College of Surgeons in Ireland, Dublin, Ireland; 47 Royal Women’s Hospital Melbourne, Melbourne, Australia; 48 Royal College of Surgeons in Ireland, Dublin, Ireland; 49 Department of Obstetrics and Gynaecology, Rotunda Hospital Dublin, Dublin, Ireland; 50 Department of Neonatology, Children’s Minnesota, Minneapolis; 51 Department of Obstetrics, Birmingham Women’s and Children’s NHS Foundation Trust, Birmingham, United Kingdom; 52 Department of Obstetrics & Gynaecology, The Chinese University of Hong Kong, Hong Kong, China; 53 Department of Clinical Epidemiology, Leiden University Medical Center, Leiden, the Netherlands; 54 Department of Hematology, Leiden University Medical Center, Leiden, the Netherlands

## Abstract

**Importance:**

Preventive efforts in pregnancy-related alloimmunization have considerably decreased the prevalence of hemolytic disease of the fetus and newborn (HDFN). International studies are therefore essential to obtain a deeper understanding of the postnatal management and outcomes of HDFN. Taken together with numerous treatment options, large practice variations among centers may exist.

**Objectives:**

To assess variations in postnatal management and outcomes of HDFN among international centers and to identify opportunities to improve care.

**Design, Setting, and Participants:**

In this international, retrospective, cohort study, 31 expert centers from 22 countries retrieved data on neonates with HDFN managed between January 1, 2006, and July 1, 2021. Statistical analysis was performed from July 19, 2023, to October 28, 2024.

**Main Outcomes and Measures:**

Main outcomes included the frequency of exchange transfusions, administration of intravenous immunoglobulin, administration of erythropoiesis-stimulating agents, and red blood cell transfusions, as well as the association of gestational age at birth with exchange transfusion frequency and risk factors for adverse neonatal outcomes.

**Results:**

The study included 1855 neonates (median gestational age at birth, 36.4 weeks [IQR, 35.0-37.3 weeks]; 1034 boys [55.7%]), of whom 1017 (54.8%) received any form of antenatal treatment. Most neonates (1447 [78.0%]) had anti-D antibodies. Exchange transfusions were performed in 436 neonates (23.5%), with proportions in exchange transfusion frequency varying from 0% to 78% among centers. Intravenous immunoglobulin was administered to 429 of 1743 neonates (24.6%), with proportions varying from 0% to 100% among centers. A higher gestational age at birth was associated with a reduction in exchange transfusion frequency in neonates with intrauterine transfusion, decreasing from approximately 38.2% (13 of 34) at 34 weeks to 16.8% (18 of 107) after 37 weeks and 0 days. A weekly increase in gestational age at birth was associated with a 43.3% decrease (95% CI, 36.1%-49.7%) in the likelihood of adverse neonatal outcomes, and neonates who received an exchange transfusion were 1.55 (95% CI, 1.10-2.18) times more likely to experience unfavorable outcomes.

**Conclusions and Relevance:**

In this cohort study of neonates with HDFN managed at 31 centers in 22 countries, significant practice variations in the postnatal management of HDFN were identified, highlighting the lack of, and need for, consensus. The study suggests that there is a potential beneficial clinical association of waiting for delivery until after 37 weeks and 0 days with frequency of exchange transfusions among neonates with HDFN. The framework to implement international guidelines is provided.

## Key Points

**Question:**

Do postnatal management and outcomes of hemolytic disease of the fetus and newborn (HDFN) vary internationally?

**Findings:**

This cohort study with1855 neonates found large variations in exchange transfusion frequency and use of intravenous immunoglobulins. Higher gestational age at birth was associated with a reduction in exchange transfusion frequency and fewer unfavorable outcomes.

**Meaning:**

This study suggests that there are considerable variations in postnatal management and outcomes of HDFN among centers worldwide and that there is a potential positive association of delivery after 37 weeks and 0 days with exchange transfusions in HDFN, providing a foundation to achieve consensus and implement international guidelines.

## Teaser

This cohort study assesses variations in postnatal management and outcomes of hemolytic disease of the fetus and newborn among international centers and evaluates opportunities to improve care.

## Introduction

Preventive efforts in pregnancy-related alloimmunization have considerably decreased the prevalence of hemolytic disease of the fetus and newborn (HDFN).^1^ In HDFN, maternal immunoglobulin G (IgG) directed against blood group antigens destroys fetal red blood cells (RBCs).^1,2,3^ Fetal anemia may result in hydrops fetalis and perinatal death. Antenatal management focuses on identifying at-risk pregnancies and treating anemia with intrauterine transfusions (IUTs).^3^ After birth, management focuses on treating hyperbilirubinemia^2^ to prevent kernicterus.^4^ The cornerstone of treating hyperbilirubinemia is intensive phototherapy and exchange transfusions (ETs).^5^ Intravenous immunoglobulins (IVIGs) may be used to restrict hyperbilirubinemia severity, in spite of limited evidence of their efficacy.^6^ In addition, RBC transfusions may be required due to ongoing hemolysis and hyporegenerative anemia.^7^

Given the rarity of HDFN, large differences in postnatal management and outcomes may exist among centers. International collaborations are essential to obtain a deeper understanding of differences in postnatal management and outcomes of HDFN among centers and to identify opportunities for guideline changes. We aimed to describe practice variations and outcomes in postnatal management of HDFN to evaluate the association of gestational age (GA) at birth with ET frequency and to examine risk factors for adverse neonatal outcomes.

## Methods

### Study Design, Population, and Data Collection

The Leiden University Medical Center (LUMC) initiated this international, retrospective cohort study (Worldwide Collaboration for Hemolytic Disease of the Fetus and Newborn [DIONYSUS] study) that was approved by the medical ethical committee of Leiden-Delft-Den Haag, adhered to the principles of the Declaration of Helsinki,^8^ and complied with the General Data Protection Regulation. Local investigators obtained approval from institutional review boards or ethical committees, and written informed consent was obtained when necessary. A total of 31 centers from 22 countries participated (eTable 1 in Supplement 1). We included all patients with HDFN who were cared for between January 1, 2006, and July 1, 2021. Center names are pseudonymized for privacy and confidentiality. The study adhered to the Strengthening the Reporting of Observational Studies in Epidemiology (STROBE) reporting guideline.

We collected data from pregnancies that resulted in perinatal death (≥16 weeks and 0 days), from pregnancies with liveborn neonates who received antenatal treatment with IUT and/or IVIG, and from pregnancies with liveborn neonates without antenatal treatment who were treated with intensive phototherapy, ET, or RBC transfusions. Local investigators reviewed medical records, assessed eligibility, and entered data in an online Castor EDC electronic case report form (eCRF). The eCRF contained built-in data validation. Remaining inconsistencies were checked with local investigators. We collected data on serologic characteristics, referral, obstetric history, antenatal management, delivery, postnatal management, and neonatal morbidity and mortality. Center-specific postnatal practices are displayed in eTable 2 in Supplement 1.

This article describes postnatal practice variations and outcomes. Antenatal outcomes are described in a separate article.^9^

### Assessment of Postnatal Management

Frequencies of postnatal treatment were described as part of all included neonates and per center. Red blood cell transfusion frequency was depicted as the proportion of neonates who received 1 or more RBC transfusions during initial admission among neonates with data available on admission duration and timing of RBC transfusions.

### Statistical Analysis

Statistical analysis was performed from July 19, 2023, to October 28, 2024. The statistical analysis plan was finalized prior to analyses, in collaboration with the department of epidemiology at the LUMC. During the finalization of the article and peer review of the study, certain aspects of the statistical analysis plan were reevaluated, leading to a more descriptive approach in the presentation of findings. Continuous data are presented as median (IQR) values and categorical data as proportions. Statistical analyses were performed using SPSS Statistics, version 28.0 (IBM Corp).

To quantify the association of GA at birth with ET frequency, we described ET frequencies per week of GA supplemented by the Kruskall-Wallis test for independent samples to statistically assess differences in the proportion of neonates with ET per GA at birth. For this analysis, we selected singleton neonates with primarily anti-D–mediated or anti-c–mediated HDFN born at a GA of 33 weeks and 0 days or more. To prevent potential confounding on the GA at birth or the need for an ET in this analysis,^10^ we excluded cases with antenatal IVIG administration, antenatal plasmapheresis, severe fetal hydrops at first IUT (defined as severe ascites, pericardial effusion, pleural effusion, or skin edema) or hydrops at birth, or postnatal IVIG administration. Five subgroups were formed based on GA at birth: 33 weeks and 0 days to 33 weeks and 6 days, 34 weeks and 0 days to 34 weeks and 6 days, 35 weeks and 0 days to 35 weeks and 6 days, 36 weeks and 0 days to 36 weeks and 6 days, and 37 weeks or more.

We assessed the association between potential risk factors and adverse neonatal outcomes by performing a univariate and multivariate logistic regression analysis using the following independent variables: antenatal treatment with IUTs, number of IUTs, severe hydrops at first IUT, GA at birth, birth weight, hemoglobin level at birth, and whether an ET was performed. Multicollinearity between independent variables that were statistically significant in multivariate logistic regression analysis was assessed through interaction terms and variance inflation factors. The dependent variable, adverse neonatal outcome, was defined as a composite outcome consisting of 1 or more of the following: respiratory distress syndrome (RDS), defined as respiratory failure receiving mechanical ventilation and/or surfactant and confirmed by chest radiograph; necrotizing enterocolitis (NEC) at Bell stage 2A or higher^11^; culture-proven bacterial sepsis, defined as clinical illness and a positive blood culture; severe cerebral injury, defined as cystic periventricular leukomalacia of grade 2 or higher,^12^ intraventricular hemorrhage of grade 3 or higher,^13^ ventricular dilatation larger than the 97th percentile, arterial or venous infarct, or porencephalic or parenchymal cysts; kernicterus^4^; and neonatal mortality, defined as death of a liveborn infant 28 days or less after birth, regardless of GA. All *P* values were from 2-sided tests and results were deemed statistically significant at *P* < .05.

## Results

### Patient Inclusion

A total of 2443 pregnancies were registered, of which 23 (0.9%) were excluded for missing antenatal data, 95 pregnancies (3.9%) resulted in perinatal death, and 470 pregnancies (19.2%) with a liveborn neonate had missing data regarding postnatal treatment. Baseline characteristics of excluded cases are reported in eTable 3 in Supplement 1. We thus included 1855 of 2443 neonates (75.9%) for analyses (Table). Of the 1855 included neonates (median GA at birth, 36.4 weeks [IQR, 35.0-37.3 weeks]; 1034 boys [55.7%] and 821 girls [44.3%]), 1017 (54.8%) received any form of antenatal treatment, and 838 (45.2%) received no antenatal treatment but were treated with intensive phototherapy, ET, or RBC transfusions. Most neonates (1447 [78.0%]) had anti-D antibodies. eTable 4 in Supplement 1 shows a detailed overview of the alloantibodies found among the included cohort.

**Table.** Baseline Clinical Characteristics of Included Liveborn Cases With Antenatal Treatment and Liveborn Cases Managed Without Antenatal Treatment

Characteristic	No. (%)	
	Liveborn, with any form of antenatal treatment (n = 1017)	Liveborn, no antenatal treatment (n = 838)
Primary alloantibody		
Anti-D	785 (77.2)	662 (79.0)
Anti-K1 (Kell)	133 (13.1)	20 (2.4)
Other	99 (9.7)	156 (18.6)
Gravidity, median (IQR)	3 (2-4)	3 (2-4)
Parity, median (IQR)	2 (1-3)	1 (1-2)
IUT only	948 (93.2)	NA
IVIG and IUT	39 (3.8)	NA
IVIG, plasmapheresis, and IUT	17 (1.7)	NA
IVIG only	11 (1.1)	NA
IVIG and plasmapheresis only	2 (0.2)	NA
Cesarean delivery	597 (58.7)	363 (43.3)
Gestational age at birth, median (IQR), wk	35.7 (34.3-36.7)	37.1 (36.1-38.0)
Birth weight, median (IQR), g	2665 (2310-2995)	2960 (2599-3286)
Sex		
Female	485 (47.7)	336 (40.1)
Male	532 (52.3)	502 (59.9)
Hemoglobin level at birth, median (IQR), g/dL	12.4 (10.4-14.3)	14.2 (11.7-16.3)
Intensive phototherapy	877 (96.9)^a^	833 (99.4)
Days with intensive phototherapy, median (IQR)	4 (2-5)^a^	4 (3-6)
Exchange transfusions	247 (24.3)	189 (22.6)
Number of exchange transfusions, median (IQR)	1 (1-1)	1 (1-1)
Postnatal IVIG	238 (26.3)^a^	191 (22.8)
RBC transfusions during first admission^b^	185 (62.5)^a^	143 (77.7)

Abbreviations: IVIG, intravenous immunoglobulin; IUT, intrauterine transfusion; NA, not applicable; RBC, red blood cell.

SI conversion factors: To convert hemoglobin to grams per liter, multiply by 10.0.

^a^ Data were unknown in 1 center, accounting for 112 neonates with antenatal treatment; percentages were therefore calculated using a total of 905 neonates with available data on the specific variables.

^b^ Among 296 neonates with antenatal treatment and 184 neonates without antenatal treatment that have data available on duration of admission and timing of RBC transfusions.

### Postnatal Treatment and Complications

#### Intensive Phototherapy

Of 1743 of 1855 neonates (94.0%) with data on intensive phototherapy, 1710 (98.1%) received phototherapy, with a median duration of 4 days (IQR, 3-6 days) (Table). Of the remaining 33 neonates without intensive phototherapy, 28 received 1 or more RBC transfusion, and 5 treated antenatally with IUTs received erythropoiesis-stimulating agents (ESAs). Complications associated with phototherapy occurred in 29 of 1743 neonates (1.7%) and included 15 neonates with hypothermia (0.9%), 7 with hyperthermia (0.4%), 4 with bronze baby syndrome (0.2%), and 3 with skin rash or pustulosis (0.2%).

#### Exchange Transfusions

**Figure 1.**
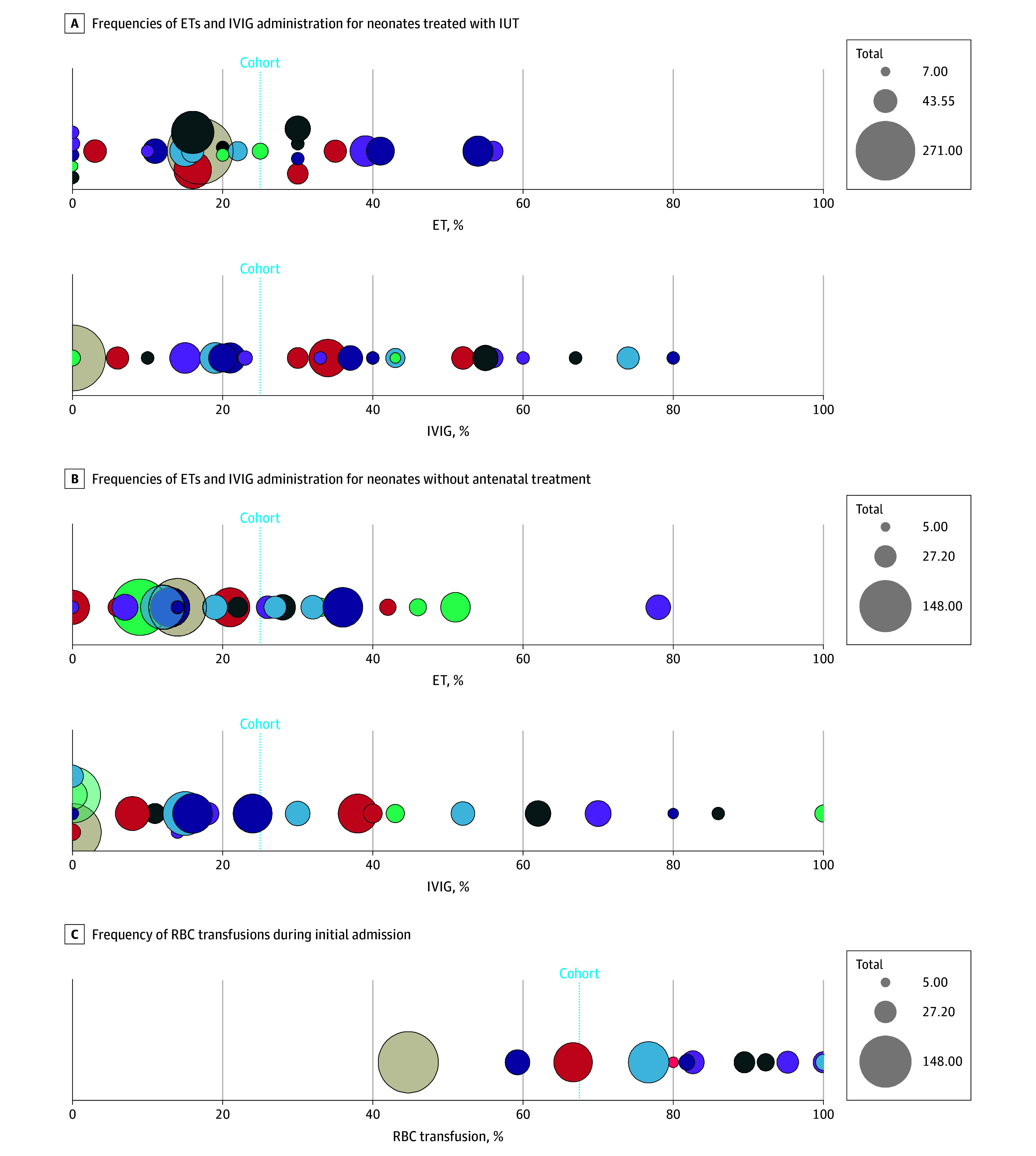
Bubble Plots of Treatment Frequencies Per Center A, Frequencies among centers of exchange transfusions (ETs) and intravenous immunoglobulin (IVIG) administration among neonates treated with intrauterine transfusion (IUT). B, Frequencies among centers of ETs and IVIG administration among neonates without antenatal treatment. C, Red blood cell (RBC) transfusions during initial admission. Each bubble represents a center. The size of the bubble indicates the number of cases.

A total of 436 of 1855 neonates (23.5%) received 1 or more ETs, with proportions ranging between 0% and 78% among centers (Figure 1A and B). Exchange transfusion frequencies were similar between neonates with and neonates without antenatal treatment (24.2% [246 of 1017] and 22.5% [189 of 839], respectively). The total number of ETs was known for 404 of 435 neonates (92.9%), of whom 331 (81.9%) needed only 1 ET, while 50 (12.4%) required 2 ETs and 23 (5.7%) received 3 ETs or more. A total of 530 ETs were performed. Procedure-specific data were available for 363 of 530 ETs (68.5%) in 325 neonates. Complications occurred in 57.0% of procedures (207 of 363), including thrombocytopenia (platelets, <100 × 10^3^/µL [to convert to cells ×10^9^ per liter, multiply by 1.0]) (198 of 363 [54.5%]), leukocytopenia (leukocytes, <5000/µL [to convert to cells ×10^9^ per liter, multiply by 0.001]) (52 of 363 [14.3%]), hypocalcemia (calcium, <80 mg/dL [to convert to millimoles per liter, multiply by 0.25]) (25 of 363 [6.9%]), culture-positive bacterial sepsis (6 of 363 [1.7%]), and hyperkalemia (potassium, >65 mEq/L [to convert to millimoles per liter, multiply by 1.0]) (4 of 363 [1.1%]). No deaths due to ETs were reported.

#### Intravenous Immunoglobulins

Of 1743 of 1855 neonates (94.0%) with data on IVIG administration, 429 (24.6%) received IVIG, with proportions ranging between 0% and 100% among centers (Figure 1A and B). Overall, 23 of 31 centers (74.2%) administered IVIG to 1 or more neonates (eTable 2 in Supplement 1). In these centers, IVIG was provided to neonates with severe hyperbilirubinemia to delay or prevent an impending ET. Information on total IVIG dosing received was available for 337 of 429 neonates (78.6%). Two of 337 neonates (0.6%) received less than 0.5 g/kg of IVIG, 39 (11.6%) received 0.5 g/kg, 44 (13.1%) received 0.75 g/kg, 132 (39.2%) received 1 g/kg, and 120 (35.6%) received more than 1 g/kg. Among 409 of 429 neonates (95.3%) with information on number of infusions, 260 (63.6%) received 1 infusion, 120 (29.3%) received 2 infusions, and 29 (7.1%) received 3 or more infusions. Of 429 neonates treated with IVIG, 1 (0.2%) born at 37 weeks and 0 days developed NEC that was managed conservatively. No further complications associated with IVIG were reported.

#### RBC Transfusions

Duration of admission and data on exact timing of RBC transfusions were known for 465 of 1855 neonates (25.1%) from 14 of 31 centers (45.2%). Among these, 314 neonates (67.5%) were treated with 1 or more RBC transfusions during initial admission, ranging from 44.7% up to 100% among centers (Figure 1C).

Data on pretransfusion hemoglobin levels were available for 1211 of 1565 RBC transfusions (77.4%) given to 634 of 808 neonates (78.5%) managed at 13 centers. The median pretransfusion hemoglobin level was 7.9 g/dL (IQR, 7.1-8.9 g/dL), ranging between a median of 7.6 g/dL and a median of 12.4 g/dL among centers (to convert to grams per liter, multiply by 10.0) (eFigure 1 in Supplement 1). Data on complications were available for 1258 of 1565 RBC transfusions (80.4%). Complications occurred in 3 of 1258 RBC transfusions (0.2%) and included 2 neonates reported to have transfusion-associated NEC and 1 neonate with hypernatremia and hypoalbuminemia.

### Erythropoiesis-Stimulating Agents

A total of 118 of 1855 neonates (6.4%) received ESAs, with proportions ranging between 0% and 47.3% among centers. Of these 118 neonates, 72 (61.0%) received an IUT. Overall, 13 of 31 centers (41.9%) provided ESAs to at least 1 neonate, of which 1 center provided ESAs as part of a randomized clinical trial. Data on dosage and timing of administration were not retrieved.

### GA at Birth and ET Frequency

Of 1855 eligible neonates, 990 (53.4%) met the criteria for the comparative analysis to quantify the association between GA at birth and ET frequency (eFigure 2 and eTables 5 and 6 in Supplement 1). Thus, 865 of 1855 neonates (46.6%) were excluded from this analysis due to the presence of previously mentioned potential confounders that could affect GA at birth or the need for an ET.

**Figure 2.**
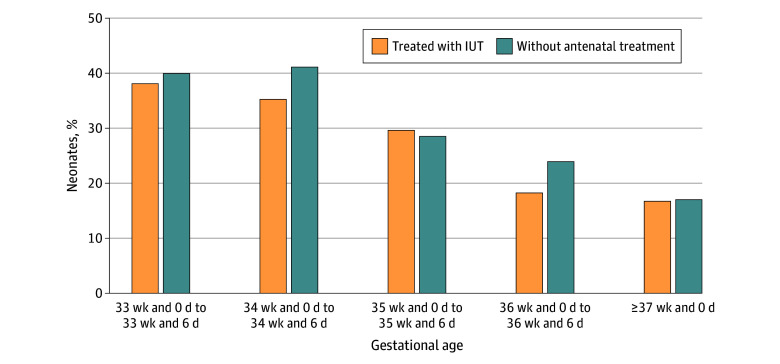
Exchange Transfusion Frequency by Gestational Age Shown is the exchange transfusion frequency in neonates treated with intrauterine transfusion (IUT) and neonates without antenatal treatment, by gestational age.

Among 441 neonates with 1 or more IUTs included in this analysis, the ET frequency decreased with increasing GA at birth from 38.2% (13 of 34) in week 33 and 0 days to week 33 and 6 days down to 16.8% (18 of 107) in week 37 and 0 days or later (Figure 2). Among these neonates, the ET frequency was higher in week 34 and 0 days to week 34 and 6 days compared with week 37 and 0 days or later (test statistic = 40.638, *df* = 4; *P* = .009) and was higher in week 35 and 0 days to week 35 and 6 days compared with week 37 and 0 days or later (test statistic = 26.159, *df* = 4; *P* = .047).

We also found decreasing ET frequencies with increasing GA at birth among 549 neonates without antenatal treatment, ranging from 40.0% (2 of 5) in week 33 and 0 days to week 33 and 6 days down to 17.1% (66 of 386) in week 37 and 0 days or later. The ET frequency was lower among neonates without antenatal treatment born at 37 weeks and 0 days or later compared with those born at week 34 and 0 days to week 34 and 6 days (test statistic = 73.025, *df* = 4; *P* = .01) and week 35 and 0 days to week 35 and 6 days (test statistic = 31.436, *df* = 4; *P* = .047).

### Risk Factors for Adverse Neonatal Outcomes

**Figure 3.**
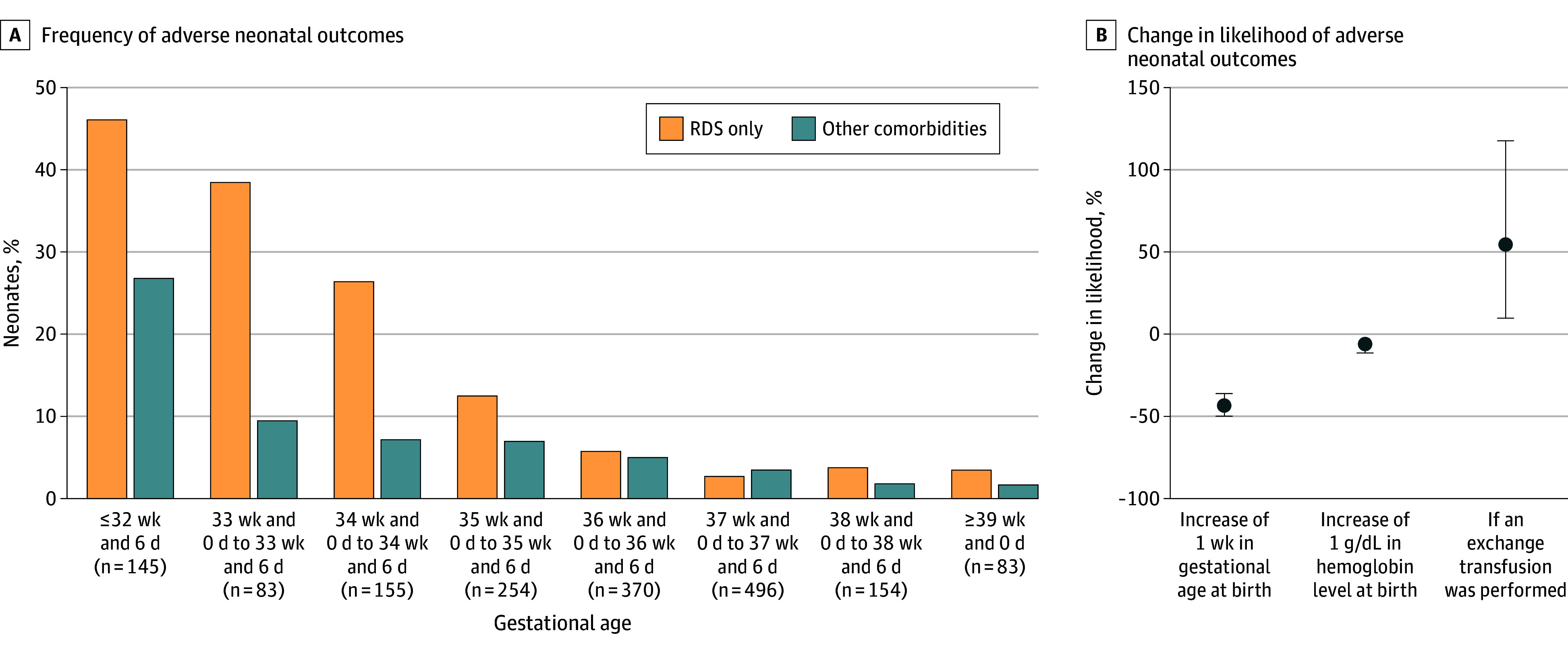
Adverse Neonatal Outcomes by Gestational Age and Association of Variables With Likelihood of Adverse Neonatal Outcomes A, Frequency of adverse neonatal outcomes by gestational age at birth. B, Percentage change in the likelihood of adverse neonatal outcomes. Error bars indicate 95% CIs. RDS indicates respiratory distress syndrome. To convert hemoglobin to grams per liter, multiply by 10.0.

Among 1740 of 1855 neonates (93.8%) with data available, adverse neonatal outcomes were present in 332 (19.1%); of these, 216 (65.1%) showed RDS only (Figure 3A). The remaining 116 neonates showed a combination of 178 reported morbidities: RDS (n = 46), culture-positive bacterial sepsis (n = 58), NEC (n = 27), severe cerebral injury (n = 21), kernicterus (n = 6), and death (n = 20) (eFigure 3 in Supplement 1). We found decreasing rates of neonatal morbidities, with the exception of kernicterus, with increasing GA at birth.

A total of 1552 of 1855 neonates (83.7%) had complete information on potential risk factors for adverse neonatal outcomes and were included in the exploratory analysis. We found that every additional week of GA at birth was associated with a 43.3% (95% CI, 36.1%-49.7%) decrease in the likelihood of adverse neonatal outcomes (*P* < .001) (Figure 3B). Similarly, an increase of 1 g/dL in hemoglobin level at birth was associated with a decrease in the likelihood of adverse neonatal outcomes of 5.9% (95% CI, 2.3%-11.3%; *P* = .01). In addition, neonates who received an ET were 1.55 (95% CI, 1.10-2.18) times more likely to experience adverse neonatal outcomes (*P* = .01) compared with neonates without ET. The model demonstrated a Nagelkerke *R*^2^ value of 0.345. Interaction terms among GA at birth, hemoglobin level at birth, and the need for an ET were statistically nonsignificant, and variance inflation factors revealed no multicollinearity (eTable 7 in Supplement 1). Treatment with IUT, number of IUTs, severe hydrops at first IUT, and birth weight were not associated with adverse neonatal outcomes.

### Mortality

Death occurred among 20 of 1855 neonates (1.1%), 12 of whom received antenatal treatment with IUT. Hemolytic disease of the fetus and newborn was caused primarily by anti-D (n = 16), anti-c (n = 2), anti-K (n = 1), and anti-C (n = 1). The median GA at birth among neonates who died was 30.6 weeks (IQR, 28.7-32.9 weeks), and the median hemoglobin level at birth was 10.0 g/dL (IQR, 7.8-13.9 g/dL). Death occurred at a median of 12 days (IQR, 6-23 days) after birth. Cause of death was known for 18 neonates and included sepsis (n = 6), multiorgan failure (n = 5), NEC (n = 2), RDS (n = 1), abdominal surgery complications (n = 1), hydrops (n = 1), hydrops and suspected gestational alloimmune liver disease (n = 1), and severe anemia after unsuccessful percutaneous umbilical cord sampling (n = 1). Hydrops was present among 9 of 20 neonates (45.0%) born at a median GA of 31.3 (IQR, 25.8-32.3), of whom 7 received 1 or more IUTs.

## Discussion

This international multicenter cohort study shows considerable variability in ET frequency, use of IVIG, use of ESA, and RBC transfusions among centers treating neonates with HDFN. A higher GA at birth was associated with a lower ET frequency, and we found a lower occurrence of adverse neonatal outcomes among neonates born at a higher GA, among neonates with a higher hemoglobin level at birth, and among neonates with no ET.

A literature review showed large variations in ET rates in HDFN among published studies.^5^ However, those analyses were limited by few high-quality studies, low case numbers, and few studies from middle- and low-income countries.^5^

To our knowledge, this is the first study to extensively assess the frequency of treatment options for neonates with HDFN in an international setting. We found that 23.5% of neonates received an ET, but differences among centers were high, with proportions varying from 0% to 78%. These large variations may be explained by variability in the application of intensive phototherapy^14,15^ or lack of effective phototherapy devices.^15^ Also, differences in bilirubin thresholds among different ET protocols may be of influence. Last, it may be due to chance that some centers may have cared for relatively more neonates for whom an ET was required.

We also found that 24.6% of neonates received IVIG, but some centers never used IVIG while other centers administered IVIG to all neonates. Intravenous immunoglobulin is thought to inhibit hemolysis by blocking Fc receptors, enhancing antibody clearance, and lowering bilirubin levels.^16^ However, based on current literature, the role of IVIG in such situations remains unclear. Our findings highlight the need for a randomized clinical trial to assess the potential effect of IVIG in preventing the need for ET.^6,17^ We found only 1 neonate who developed NEC among more than 429 neonates treated with IVIG, which is in contrast with a study reporting 10 cases of NEC among 167 neonates treated with IVIG.^18^ That study was, however, limited by an unclear definition of NEC.^19^ The findings from this study may reduce hesitancy among neonatologists in administering IVIG, although the potential benefit of IVIG to prevent an impending ET has not been quantified.^6,19,20^

Additionally, we found considerable differences in the proportion of neonates who received RBC transfusions during initial admission and in hemoglobin levels prior to transfusions. Monitoring for late anemia is essential as it may occur up to 3 months after birth, with 60% to 90% of neonates needing a median of 2 transfusions.^5,7^ Due to a lack of data after discharge, we were unable to determine variations in postdischarge management. This may be due to monitoring performed elsewhere, but it may also be due to a lack of awareness.

Our study also shows large variability among centers in deciding when to deliver neonates with HDFN. Inducing preterm delivery (<37 weeks and 0 days) in high-risk pregnancies is widely accepted to limit the potential effect of increasing IgG transport with increasing GA. However, this policy of preterm delivery comes at a trade-off for decreased fetal maturation and may pose risks for severe hyperbilirubinemia and a higher need for ETs. Studies to quantify the association of different policies on induction at different GAs with the frequency of ETs have been challenging due to the rarity of HDFN. This study has enabled us to approach this issue. We have shown a lower ET frequency among neonates born at week 37 and 0 days or later compared with earlier GAs at birth, supporting the policy of full-term delivery. The hypothesis is that increased fetal maturation in full-term neonates may improve bilirubin conjugation compared with preterm neonates. Also, we found that hemoglobin levels at birth were comparable between groups, despite the continued or even increased transport of maternal IgG at higher GAs.^21^ Although it is difficult to eliminate unmeasured confounding factors in observational data, we were able to adequately adjust the analysis for measured potential confounding factors (ie, antenatal treatment with IVIG or plasmapheresis, severe hydrops at first IUT, hydrops at birth, and postnatal IVIG administration) through this joint data collection. Conversely, controlling for potential confounders might limit the generalizability of these results.

In addition, we identified GA at birth, hemoglobin level at birth, and treatment with ET as factors associated with a significant change in the likelihood of adverse neonatal outcomes. Although an increase of 1 g/dL in hemoglobin level at birth was statistically significant, an increase of 1 week in GA at birth and the performance of an ET were associated with a considerably larger change in the likelihood of adverse neonatal outcomes. It is therefore imperative that clinicians focus on deliveries at a later GA and prevent the need for ETs.

### Strengths and Limitations

This study has some strengths, including the involvement of several participating centers from many countries, consequently increasing the generalizability of our findings to a broader population. By combining retrospective data from patients with HDFN, we were able to gather clinical data in a cost- and time-efficient manner, enabling us to study this rare disease in a large population.

This study also has some limitations. Its retrospective design limits our ability to establish causality. Variations in management of HDFN may partly be explained by the chance that some centers cared for a higher proportion of severely ill neonates. Nevertheless, the use of data spanning a 15-year period may help minimize this possibility.

## Conclusions

This cohort study of neonates with HDFN cared for at 31 centers in 22 countries demonstrates significant variability in ETs, IVIGs, and RBC transfusions among centers. Variations in guidelines, insufficient evidence, limited awareness, or obstacles in implementing procedures may underlie these differences. We found that increased GA at birth was associated with a lower ET frequency and a lower likelihood of adverse neonatal outcomes. These findings highlight the potential positive clinical characteristics associated with delaying delivery until after 37 weeks and 0 days weeks as well as an opportunity to implement international guidelines.

## Notes

Supplement 1. **eTable 1.** Alphabetical Outline of All Collaborating Centers and Investigators **eTable 2.** Postnatal Practice Characteristics per Center **eTable 3.** Baseline Characteristics of Excluded Cases **eTable 4.** Distribution of Alloantibodies **eFigure 1.** Pre-Transfusion Hemoglobin Levels per Center **eFigure 2.** Flowchart of Inclusion and Exclusion in Assessing the Effect of Gestational Age at Birth on Exchange Transfusion Frequency **eTable 5.** Baseline Characteristics of Neonates With One or More Intrauterine Transfusions Included in Analysis on Gestational Age at Birth and Exchange Transfusions Frequency **eTable 6.** Baseline Characteristics of Neonates Without Antenatal Treatment Included in Analysis on Gestational Age at Birth and Exchange Transfusions Frequency **eTable 7.** Variance Inflation Factors of Independent Variables Included in the Analysis on Adverse Neonatal Outcome **eFigure 3.** Frequency of Neonatal Comorbidities and Mortality per Gestational Age at Birth

Supplement 2. **Nonauthor Collaborators.** Worldwide Collaboration for Hemolytic Disease of the Fetus and Newborn (DIONYSUS) Investigators

Supplement 3.
Data Sharing Statement
