# Supplementary material for: Variations and Opportunities in Postnatal Management of Hemolytic Disease of the Fetus and Newborn
Source: JAMA Netw Open. 2025 Jan 10;8(1):e2454330. doi: 10.1001/jamanetworkopen.2024.54330 (PMC11724339; doi:10.1001/jamanetworkopen.2024.54330)
Supplement: Supplement 1. — eTable 1. Alphabetical Outline of All Collaborating Centers and Investigators eTable 2. Postnatal Practice Characteristics per Center eTable 3. Baseline Characteristics of Excluded Cases eTable 4. Distribution of Alloantibodies eFigure 1. Pre-Transfusion Hemoglobin Levels per Center eFigure 2. Flowchart of Inclusion and Exclusion in Assessing the Effect of Gestational Age at Birth on Exchange Transfusion Frequency eTable 5. Baseline Characteristics of Neonates With One or More Intrauterine Transfusions Included in Analysis on Gestational Age at Birth and Exchange Transfusions Frequency eTable 6. Baseline Characteristics of Neonates Without Antenatal Treatment Included in Analysis on Gestational Age at Birth and Exchange Transfusions Frequency eTable 7. Variance Inflation Factors of Independent Variables Included in the Analysis on Adverse Neonatal Outcome eFigure 3. Frequency of Neonatal Comorbidities and Mortality per Gestational Age at Birth [file jamanetwopen-e2454330-s001.pdf]

Supplementary Online Content

de Winter DP, Verweij EJT, Debeer A, et al; Worldwide Collaboration for Hemolytic Disease of the Fetus and Newborn (DIONYSUS) Investigators. Variations and opportunities in postnatal management of hemolytic disease of the fetus and newborn. *JAMA Netw Open.* 2025;8(1):e2454330. doi:10.1001/jamanetworkopen.2024.54330

eTable 1. Alphabetical Outline of All Collaborating Centers and Investigators

eTable 2. Postnatal Practice Characteristics per Center

eTable 3. Baseline Characteristics of Excluded Cases

eTable 4. Distribution of Alloantibodies

eFigure 1. Pre-Transfusion Hemoglobin Levels per Center

eFigure 2. Flowchart of Inclusion and Exclusion in Assessing the Effect of Gestational Age at Birth on Exchange Transfusion Frequency

eTable 5. Baseline Characteristics of Neonates With One or More Intrauterine Transfusions Included in Analysis on Gestational Age at Birth and Exchange Transfusions Frequency

eTable 6. Baseline Characteristics of Neonates Without Antenatal Treatment Included in Analysis on Gestational Age at Birth and Exchange Transfusions Frequency

eTable 7. Variance Inflation Factors of Independent Variables Included in the Analysis on Adverse Neonatal Outcome

eFigure 3. Frequency of Neonatal Comorbidities and Mortality per Gestational Age at Birth

This supplementary material has been provided by the authors to give readers additional information about their work.

© 2025 de Winter DP et al. *JAMA Network Open.*

eTable 1. Alphabetical Outline of All Collaborating Centers and Investigators

eTable 1 provides an outline of all participating centers, its location and country and the investigators associated.

A total of 46 centers were invited by the initiating center to participate in the study, of these 30 centers participated totaling at 31 participating centers. Of the 16 remaining centers, 14 did not respond to the invitation or did not have an interest in participating, 1 center was unable to achieve judicial and ethical approval within the study time frame and 1 centers' participation was put on hold by the Dutch Federation of Medical Universities due to an ongoing conflict with Ukraine.

Center	City and country	Investigators
Birmingham Women's and Children's NHS Foundation Trust	Birmingham, United Kingdom	Wing Ting Tse, James Castleman, Mark Kilby, Rob Negrine
Buzzi Children's Hospital	Milan, Italy	Francesco Cavigioli, Sofia Fatima Giuseppina Colombo, Francesca Castoldi, Chiara Nava
Centre Hospitalier Universitaire de Lille	Lille, France	Kévin Le Duc, Louise Ghesquiere, Baptiste Teillet, Thameur Rakza
Charité-Universitätsmedizin Berlin	Berlin, Germany	Christof Dame, Jessica D. Blank, Stefan Verlohren, Beate Mayer
CNRHP Trousseau Hospital, Paris	Paris, France	Paul Maurice, Jean-Marie Jouannic, Marie-Gabrielle Guillemin, Agnès Mailloux
Hospital de la Mujer	La Paz, Bolivia	Ángel Guillermo Alcázar Grisi, Edgar Juan José Chávez Navarro, Mabel Laura Cabrera
Hospital de la Santa Creu i Sant Pau	Barcelona, Spain	María José Garcia Borau, Elisenda Moliner Calderon
Hospital Italiano de Buenos Aires	Buenos Aires, Argentina	Gonzalo Mariani, Maria Fernanda Galletti, Leandro Daniel Burgos Pratx
Instituto Nacional de Perinatologia	Mexico City, Mexico	Arturo Alejandro Canul-Euan, Raigam Jafet Martine-Portilla, Jose A. Montoya-Martinez
Instituto Nacional de Saúde da Mulher, da Criança e do Adolescente Fernandes Figueira (IFF/Fiocruz)	Rio de Janeiro, Brazil	Maria Cristina Pessoa dos Santos, Cynthia Amaral de Moura Sá Pacheco, Maria Elisabeth Lopes Moreira, Marcella Vasconcelos Vaena
Justus-Liebig University Gießen	Gießen, Germany	Rahel Schuler, Aline Wolter, Ivonne Bedei, Roland Axt-Fliedner
Karolinska University Hospital	Stockholm, Sweden	Kajsa Bohlin, Eleonor Tiblad, Iris Hellsing
La Paz University Hospital	Madrid, Spain	Maria Sanchez-Holgado, Aurora Viejo Llorente, Eugenia Antolin, Nieves Mendez
Leiden University Medical Center	Leiden, The Netherlands	Derek. P. de Winter, Masja de Haas, J.G. (Anske) van der Bom, E.J.T. (Joanne) Verweij, Enrico Lopriore

© 2025 de Winter DP et al. *JAMA Network Open.*

Center	City and country	Investigators
Levine Children's Hospital Atrium Healthcare Wake Forest School of Medicine	Charlotte (NC), United States of America	Matthew Saxonhouse, Ngina K Connors
Liverpool Women’s Hospital	Liverpool, United Kingdom	Borna Poljak, Asma Khalil
Medical University of Graz	Graz, Austria	Daniel Pfurtscheller, Gerhard Pichler, PhilippKlaritsch
Midwest Fetal Care Center	Minneapolis (MN), United States of America	Andrea Lampland
Ontario Fetal Centre, Mount Sinai Hospital	Toronto, Canada	Edmond Kelly, Kamini Raghuram, Johannes Keunen, Greg Ryan
Pränatal Medizin München / Kinderklinik des Klinikums Dritter Orden	München, Germany	Alexander Hohnecker
Rigshospitalet, Copenhagen University Hospital	Copenhagen, Denmark	Emilie Thorup, Olav B. Petersen, Karin Sundberg, Frederik B. Clausen
Royal College of Surgeons in Ireland / Rotunda Hospital Dublin	Dublin, Ireland	David Mackin, Fergal Malone
Shanghai First Maternity and Infant Hospital	Shanghai, People’s Republic of China	Ming Zhou, Fangfang Tao, Jiangqin Liu
Sheba Medical Center	Tel Aviv, Israel	Leah Leibovitch, Stav Cohen, Yoav Yinon, Tzipora Strauss
St George’s University Hospital	London, United Kingdom	Smriti Prasad, Asma Khalil
Tygerberg Academic Hospital, Stellenbosch University	Cape Town/Stellenbosch, South Africa	Lizelle van Wyk, Lut Geerts, Kerry Rademan
Unidade Local de Saúde de São João	Porto, Portugal	Joana Pereira-Nunes, Henrique Soares, Alexandra Matias
Universitätsklinikum Bonn	Bonn, Germany	Annegret Geipel, Johanna Rath
University Hospital KU Leuven	Leuven, Belgium	Anne Debeer, Roland Devlieger, Liesbeth Lewi, Sarah Verbeeck
University Hospitals Bristol and Weston NHS Trust	Bristol and Weston, United Kingdom	Ziju Elanjikal, Jessica Brayley
University Medical Center Ljubljana	Ljubljana, Slovenia	Jana Lozar Krivec, Aneta Soltirovska Šalamon, Erika Hrastar, Mihael Rus

© 2025 de Winter DP et al. *JAMA Network Open.*

eTable 2. Postnatal Practice Characteristics per Center

eTable 2 provides center-specific context regarding local procedures in the management of neonates affected by hemolytic disease of the fetus and newborn.

Country, City	Hyperbilirubinemia management guidelines[a]	IVIG[b]	RBC transfusion guidelines	Frequency of monitoring for late anemia	Long-term follow-up (>2 years)
Argentina, Buenos Aires	2, 3	2	National and available literature	In case of clinical symptoms	None
Austria, Graz	2, 3	3	Available literature	Once per week	In cases with exchange transfusion
Belgium, Leuven	2	4	Available literature	Once per week	None
Bolivia, La Paz	2	4	Available literature	Twice per week	In cases with exchange transfusion
Brazil, Rio de Janeiro	2	4	Institutional	Once per week	None (in all up to 1 year)
Canada, Toronto	1	2	No guidelines	Once per week	In cases with IUT and in cases with exchange transfusion
China, Shanghai	2	2	National and institutional	Once a week if admitted, once a month in out-patient clinic	In cases with exchange transfusion
Denmark, Copenhagen	5	2	Institutional	Once or twice per week depending on anemia severity	In cases with suspected neurological adverse outcome
France, Lille	4	2	Institutional	Twice per week	In cases with exchange transfusion
France, Paris	2, 4	2	National	Twice per week	In cases with IUT
Germany, Berlin	4	4	Institutional	Initially twice per week, then every 10-14 days	None
Germany, Bonn	1, 6	2	Institutional	Once per week	In cases with IUT
Germany, Gieβen	5	4	Institutional and available literature	Once per two weeks	In cases with kernicterus
Germany, München	3, 4, 5	2	Institutional and available literature	At least twice per week	In cases with critical events potentially threatening neurodevelopment
Ireland, Dublin	2, 3	2	National and institutional	Twice per week	In all cases
Israel, Tel Aviv	4	3	National	Once per week	None
Italy, Milan	2	2	Institutional	Once per week	In cases with exchange transfusion and in cases with kernicterus

© 2025 de Winter DP et al. *JAMA Network Open.*

Country, City	Hyperbilirubinemia management guidelines[a]	IVIG[b]	RBC transfusion guidelines	Frequency of monitoring for late anemia	Long-term follow-up (>2 years)
Mexico, Mexico City	2	4	Available literature	Once per week	In all cases
Netherlands, Leiden	1	4	National	Once per week	In cases with IUT
Portugal, Porto	2, 3, 4	3	National and available literature	Twice per week	In all cases
Slovenia, Ljubljana	2, 3	4	NICU guidelines	Twice per week in the first week, then every 1-2 weeks	In cases with IUT and in cases with kernicterus
South Africa, Stellenbosch	5	2	Institutional	In case of clinical symptoms	In cases with kernicterus
Spain, Barcelona	3, 7	2	Institutional	Twice per week	In cases with IUT, in cases with exchange transfusion and in cases with kernicterus
Spain, Madrid	2, 5	2	Institutional and available literature	Once per week	In cases with kernicterus
Sweden, Stockholm	4	3	Regional	First routine control 2-4 weeks after discharge, then individual decision depending on severity of HDFN, Hb level and reticulocyte count.	In cases with IUT
United Kingdom, Birmingham	3	3	National, institutional and British Society for Haematology	No protocol, based on clinical judgement	In all cases
United Kingdom, Bristol and Weston	3	2	National and institutional	Twice per week	In all cases
United Kingdom, Liverpool	3	4	National	In case of clinical symptoms	None
United Kingdom, London	3	4	National	In case of clinical symptoms	None
United States, Charlotte (NC)	2	2	Institutional and PINT data	Twice per week	In all cases
United States, Minneapolis (MN)	2	2	Institutional	Once per week	None

[a] 1 = American Academy of Pediatrics 2004, 2 = American Academy of Pediatrics 2022, 3 = NICE Guidelines 2016, 4 = national guidelines, 5 = institutional guidelines, 6 = AWMF Leitlinie der Gesellschaft für Neonatologie + pädiatrische Intensivmedizin, 7 = Bhutani, V. K., Wong, R. J., & Stevenson, D. K. (2016). Hyperbilirubinemia in Preterm Neonates. Clinics in perinatology, 43(2), 215–232. https://doi.org/10.1016/j.clp.2016.01.001
[b] 1 = To all neonates with HDFN, 2 = to all neonates with HDFN with severe hyperbilirubinemia and an impending exchange transfusion, 3 = only in rare cases, 4 = never.

© 2025 de Winter DP et al. *JAMA Network Open.*

eTable 3. Baseline Characteristics of Excluded Cases

eTable 3 provides a comparison of the baseline characteristics of the included 1855 neonates to the baseline characteristics of 470 cases that were excluded due to missing data on whether postnatal treatment was required.

	**No. (%)**	
	**Cases included (n = 1855)**	**Cases excluded (n = 470)**
Primary alloantibody		
Anti-D	1447 (78.0)	304 (64.7)
Anti-K1 (Kell)	153 (8.2)	69 (14.7)
Others	255 (13.7)	97 (20.6)
Gravidity, median [IQR]	3 [2-4]	3 [2-4]
Parity, median [IQR]	2 [1-3]	2 [1-3]
Antenatal treatment for HDFN	1017 (54.8)	332 (70.6)
Intrauterine transfusion only	948 (93.2)	311 (93.7)
IVIG and intrauterine transfusion	39 (3.8)	21 (6.3)
IVIG, plasmapheresis and IUT	17 (1.7)	0
IVIG only	11 (1.1)	0
IVIG and plasmapheresis only	2 (0.2)	0
Caesarean	960 (51.9)	217 (46.2)
Gestational age at birth, median [IQR], w	36.4 [35.0-37.3]	36.0 [33.9-37.7]
Birthweight, median [IQR], grams	2800 [2420-3130]	2740 [2265-3140]
Female	821 (44.3)	154/321 (48.0)

© 2025 de Winter DP et al. *JAMA Network Open.*

eTable 4. Distribution of Alloantibodies

eTable 4 provides an overview of the distribution of alloantibodies. The maximum known serological result of the primary antibody (the first mentioned antibody) is reported in the far right column. The timing of the maximum known serological result is unknown, it is therefore possible that higher, but thus unmeasured, results were reached.

Type of alloimmunization	Number of neonates (n = 1855)	Maximum known serological quantification of primary antibody
D	929	Unknown = 84<br>1 = 2<br>2 = 1<br>4 = 2<br>8 = 11<br>16 = 24<br>32 = 84<br>64 = 86<br>128 = 114<br>256 = 160<br>512 = 97<br>1024 = 63<br>2048 = 45<br>4096 = 35<br>8000 = 16<br>16000 = 6<br>32000 = 6<br>64000 = 4<br>120000 = 1<br><25 IU/ml = 20<br>25-50 IU/ml = 9<br>50-100 IU/ml = 10<br>>100 IU/ml = 9
D, C	358	Unknown = 13<br>8 = 3<br>16 = 1<br>32 = 16<br>64 = 20<br>128 = 45<br>256 = 51<br>512 = 47<br>1024 = 39<br>2048 = 21<br>4096 = 20<br>8000 = 11<br>16000 = 8<br>32000 = 2<br>64000 = 3<br>120000 = 1<br>≥240000 = 3<br><25 IU/ml = 5<br>25-50 IU/ml = 5<br>50-100 IU/ml = 7

© 2025 de Winter DP et al. *JAMA Network Open.*

Type of alloimmunization	Number of neonates (n = 1855)	Maximum known serological quantification of primary antibody
		>100 IU/ml = 6
K1 (Kell)	125	Unknown = 19<br>2 = 1<br>4 = 5<br>16 = 2<br>32 = 4<br>64 = 16<br>128 = 16<br>256 = 26<br>512 = 16<br>1024 = 10<br>2048 = 6<br>8000 = 2<br>32000 = 1<br>64000 = 1
c	74	Unknown = 8<br>4 = 1<br>8 = 3<br>16 = 10<br>32 = 7<br>64 = 6<br>128 = 11<br>256 = 8<br>512 = 4<br>1024 = 1<br>2048 = 2<br>4096 = 1<br><25 IU/ml = 4<br>25-50 IU/ml = 4
D, E	33	Unknown = 7<br>32 = 1<br>64 = 4<br>128 = 5<br>256 = 5<br>512 = 2<br>1024 = 1<br>2048 = 2<br>4096 = 2<br>16000 = 1<br>32000 = 1
E	29	Unknown = 9<br>4 = 2<br>16 = 3<br>32 = 4<br>64 = 4<br>128 = 2<br>256 = 3<br>1024 = 2
D, C, E	29	Unknown = 5<br>32 = 1<br>64 = 1<br>128 = 4

© 2025 de Winter DP et al. *JAMA Network Open.*

Type of alloimmunization	Number of neonates (n = 1855)	Maximum known serological quantification of primary antibody
		256 = 4<br>512 = 5<br>1024 = 2<br>2048 = 3<br>4096 = 2<br>8000 = 1<br>≥240000 = 1
E, c	24	Unknown = 4<br>8 = 1<br>16 = 2<br>32 = 1<br>64 = 5<br>128 = 5<br>256 = 5<br>512 = 1
c, E	18	Unknown = 1<br>4 = 1<br>8 = 1<br>16 = 3<br>32 = 2<br>64 = 1<br>128 = 4<br>256 = 2<br>512 = 2<br>2048 = 1
D, C, Jka	16	Unknown = 1<br>64 = 1<br>128 = 2<br>256 = 2<br>512 = 4<br>2048 = 1<br>4096 = 2<br>8000 = 1<br>16000 = 1<br>25-50 IU/ml = 1
Fya	15	Unknown = 2<br>32 = 3<br>64 = 3<br>128 = 3<br>512 = 2<br>1024 = 1<br>4096 = 1
Cw	12	Unknown = 4<br>4 = 1<br>16 = 1<br>32 = 1<br>64 = 3<br>512 = 1<br>2048 = 1
D, Jka	10	Unknown = 1<br>16 = 1<br>32 = 1

© 2025 de Winter DP et al. *JAMA Network Open.*

Type of alloimmunization	Number of neonates (n = 1855)	Maximum known serological quantification of primary antibody
		128 = 1<br>256 = 1<br>512 = 3<br>1024 = 1<br>4096 = 1
D, C, S	9	256 = 1<br>512 = 3<br>2048 = 1<br>4096 = 1<br>8000 = 1<br>16000 = 1<br>32000 = 1
C	9	Unknown = 5<br>2 = 1<br>8 = 1<br>128 = 1<br>512 = 1
M	8	Unknown = 2<br>64 = 4<br>128 = 2
D, K1	8	Unknown = 1<br>64 = 1<br>128 = 1<br>1024 = 2<br><25 IU/ml = 1<br>25-50 IU/ml = 1<br>50-100 IU/ml = 1
K1 (Kell), Jka	7	4 = 1<br>16 = 1<br>128 = 1<br>256 = 1<br>512 = 2<br>4096 = 1
D, C, Fya	7	Unknown = 1<br>32 = 1<br>64 = 1<br>256 = 1<br>512 = 1<br>2048 = 1<br>4096 = 1
U	5	256 = 3<br>512 = 1<br>1024 = 1
D, C, K1 (Kell)	5	Unknown = 2<br>64 = 1<br>512 = 2
c, K1 (Kell)	5	Unknown = 1<br>64 = 1<br>256 = 1<br>512 = 1<br>1024 = 1
Kpa	4	32 = 1

© 2025 de Winter DP et al. *JAMA Network Open.*

**Type of alloimmunization**	Number of neonates (n = 1855)	Maximum known serological quantification of primary antibody
		128 = 1<br>256 = 1<br>512 = 1
c, Jka	4	2 = 2<br>4 = 1<br>64 = 1
Jka	3	16 = 1<br>32 = 2
D, Fya	3	Unknown = 1<br>256 = 1<br>2048 = 1
D, C, M	3	64 = 1<br>64000 = 1<br><25 IU/ml = 1
C, K1 (Kell)	3	64 = 1<br>512 = 1<br>4096 = 1
c, Cw	3	Unknown = 1<br>8 = 2
Rh17	2	256 = 1<br>1024 = 1
K1 (Kell), E	2	32 = 1<br>128 = 1
e	2	16 = 1<br>128 = 1
c, M	2	4 = 1<br><25 IU/ml = 1
c, Jka, S	2	64 = 1<br><25 IU/ml = 1
c, Fya	2	64 = 1<br><25 IU/ml = 1
S	1	128 = 1
K1, S	1	512 = 1
K1 (Kell), PRIVATE	1	256 = 1
K1 (Kell), Leb2	1	128 = 1
K1 (Kell), Fya/b, Jka/b, S/s	1	1024 = 1
K1 (Kell), Fya/b, Jka/b, s	1	1024 = 1
K1 (Kell), Fya/b, Jka, s	1	2048 = 1
K1 (Kell), Fya	1	4
K1 (Kell), D, MNS1	1	256 =1
K1 (Kell), D, C, E	1	32 = 1
K1 (Kell), D, C	1	256 = 1
Jkb	1	128 = 1
Jka/b, s	1	Unknown = 1
Hro	1	512 = 1
Fyb, Jka/b, S	1	Unknown = 1
Fyb, Jka/b, s	1	Unknown = 1
Fyb, Jka, S	1	Unknown = 1
Fya, Jka/b, s	1	Unknown = 1
Fya, Jka/b	1	Unknown = 1
Fya, Jka	1	128 = 1

© 2025 de Winter DP et al. *JAMA Network Open.*

**Type of alloimmunization**	Number of neonates (n = 1855)	Maximum known serological quantification of primary antibody
Fya, E	1	256 = 1
E, K1 (Kell), Fyb	1	Unknown = 1
e, Jka, RH46	1	128 = 1
E, Jka	1	2 = 1
E, c, M	1	16 = 1
E, c, Le(a)	1	16 = 1
Doa	1	4 = 1
D, Wra	1	512 = 1
D, s	1	256 = 1
D, S	1	4096 = 1
D, M	1	32 = 1
D, Lua	1	32 = 1
D, LE1	1	256 = 1
D, Jkb	1	50-100 IU/ml = 1
D, Jka, S	1	256 = 1
D, Fyb, Jka/b, S/s	1	2048 = 1
D, Fyb, Jka/b, s	1	512 = 1
D, Fya/b, Jkb, S/s	1	32 = 1
D, Fya/b, Jka/b, S/s, M	1	512 = 1
D, Fya/b, Jka/b, S/s	1	2048 = 1
D, Fya/b, Jka/b, s, M	1	2048 = 1
D, Fya/b, Jka, S/s, M	1	2048 = 1
D, Fya, Wra	1	128 = 1
D, Fya, Jka, s	1	512 = 1
D, E, S, M	1	2048 = 1
D, E, M	1	32 = 1
D, E, Jka	1	1024 = 1
D, E, G-16	1	>100 IU/ml = 1
D, E, Fya	1	512 = 1
D, C, Wra	1	1024 = 1
D, C, SC1	1	128 = 1
D, C, Lub	1	256 = 1
D, C, Kpa	1	256 = 1
D, C, K1 (Kell), Jka	1	120000 = 1
D, C, Jkb	1	32000 = 1
D, C, Fyb, Jkb	1	Unknown = 1
D, C, Fyb, Jka, s	1	2048 = 1
D, C, Fya, Wra	1	16 = 1
D, C, Fya, S	1	2048 = 1
D, C, Fya, Jkb	1	1024 = 1
D, C, Fya, Jka, S	1	2048 = 1
D, C, E, Wra	1	32 = 1
D, C, E, S	1	4096 = 1
D, C, E, Jkb	1	128 = 1
D, C, E, Fya, Jka, S, M	1	2048 = 1
D, C, E, Fya, Jka, s	1	2048 = 1
D, C, E, Fya, Jka, S	1	512 = 1
D, c, e	1	128 = 1
Cellano (k), Wra	1	16000 = 1
Cellano (k), C	1	512 = 1
c, Wra	1	128 = 1

© 2025 de Winter DP et al. *JAMA Network Open.*

**Type of alloimmunization**	Number of neonates (n = 1855)	Maximum known serological quantification of primary antibody
c, Lub	1	32 = 1
c, Fyb	1	4096 = 1
c, Fya/b, Jka/b, s	1	1024 = 1
C, Fya	1	Unknown = 1
c, E, PRIVATE	1	16 = 1
C, E, Kpa, Kna	1	64 = 1
c, E, K1 (Kell)	1	Unknown = 1
c, E, Jka, S	1	64 = 1
c, E, Fya, M	1	<25 IU/ml = 1
c, E, Fya, Jka/b, S/s	1	64 = 1
C, E	1	Unknown = 1

© 2025 de Winter DP et al. *JAMA Network Open.*

eFigure 1. Pre-Transfusion Hemoglobin Levels per Center

eFigure 1 shows the median pre-transfusion hemoglobin levels for red blood cell transfusions per center. The pink (most left) boxplot and striped horizontal line shows the cohort median.

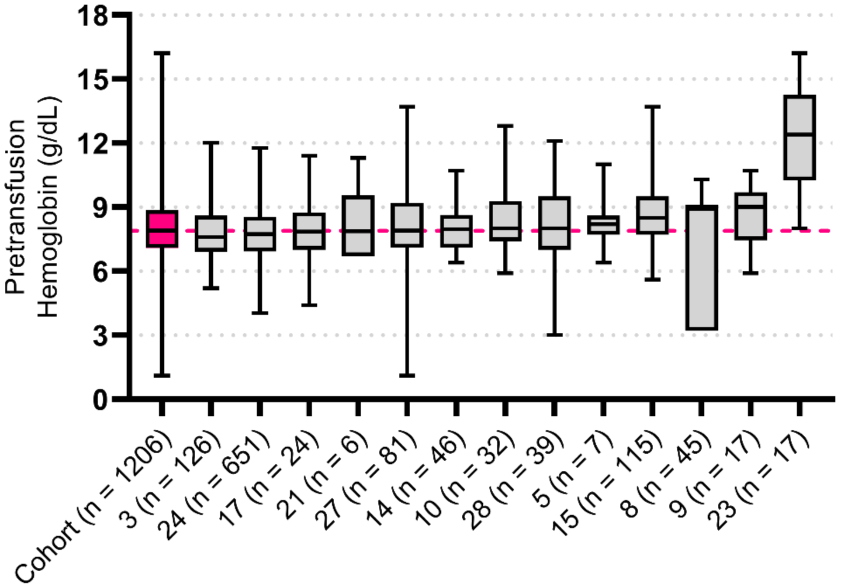

© 2025 de Winter DP et al. *JAMA Network Open.*

eFigure 2. Flowchart of Inclusion and Exclusion in Assessing the Effect of Gestational Age at Birth on Exchange Transfusion Frequency

eFigure 2 shows the flow of in- and excluding neonates in the analysis to assess the effect of gestational age at birth on the exchange transfusion frequency.

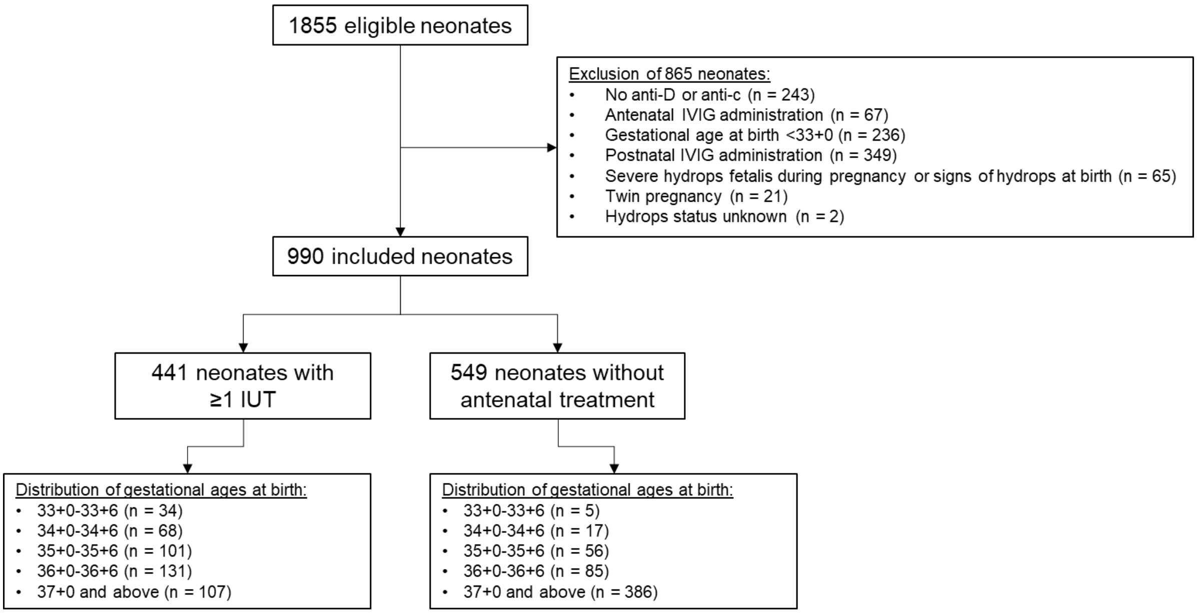

© 2025 de Winter DP et al. *JAMA Network Open.*

eTable 5. Baseline Characteristics of Neonates With One or More Intrauterine Transfusions Included in Analysis on Gestational Age at Birth and Exchange Transfusions Frequency

eTable 5: Baseline clinical characteristics of included neonates with at least one intrauterine transfusion that were in the analysis to assess the effect of gestational age at birth on the frequency of exchange transfusions.

	No. (%)				
	**33+0-33+6 (n = 34)**	**34+0-34+6 (n = 68)**	**35+0-35+6 (n = 101)**	**36+0-36+6 (n = 131)**	**≥37+0 (n = 107)**
Primary alloantibody					
Anti-D	32 (94.1)	61 (89.7)	100 (99.0)	120 (91.6)	99 (92.5)
Anti-c	2 (5.9)	7 (10.3)	1 (1.0)	11 (8.4)	8 (6.5)
Gravidity, median [IQR]	3 [2-4]	3 [2-4]	3 [2-5]	3 [2-4]	3 [2-5]
Parity, median [IQR]	2 [1-3]	2 [1-3]	2 [1-3]	1 [1-2]	2 [1-3]
Gestational age at first IUT, median [IQR], w	28.7 [25.4-31.0]	29.0 [26.4-31.9]	29.9 [25.7-32.0]	30 [27.0-32.6]	30.1 [25.6-32.6]
Number of IUTs, median [IQR]	2 [1-3]	2 [1-4]	2 [1-3]	2 [1-3]	3 [2-4]
Caesarean	31 (91.2)	58 (85.3)	57 (56.4)	45 (34.4)	39 (36.4)
Gestational age at birth, median [IQR], w	33.5 [33.3-33.8]	34.4 [34.1-34.7]	35.4 [35.1-35.7]	36.4 [36.3-36.7]	37.3 [37.1-37.6]
Birthweight, median [IQR], grams	2245 [2060-2440]	2370 [2125-2620]	2655 [2425-2945]	2852 [2618-3063]	3035 [2730-3275]
Female	17 (50.0)	31 (45.6)	53 (52.5)	72 (55.0)	53 (49.5)
Hemoglobin level at birth, median [IQR], g/dL	10.5 [7.5-14.3]	11.6 [10.0-13.5]	12.2 [10.2-15.8]	12.8 [11.4-14.3]	13.0 [11.9-14.7]
Exchange transfusion(s)	13 (38.2)	24 (35.3)	30 (29.7)	24 (18.3)	18 (16.8)

© 2025 de Winter DP et al. *JAMA Network Open.*

eTable 6. Baseline Characteristics of Neonates Without Antenatal Treatment Included in Analysis on Gestational Age at Birth and Exchange Transfusions Frequency

eTable 6: Baseline clinical characteristics of included neonates with at least one intrauterine transfusion that were included in the analysis to assess the effect of gestational age at birth on the frequency of exchange transfusions.

	No. (%)				
	**33+0-33+6 (n = 5)**	**34+0-34+6 (n = 17)**	**35+0-35+6 (n = 56)**	**36+0-36+6 (n = 85)**	**≥37+0 (n = 386)**
Primary alloantibody					
Anti-D	4 (80.0)	15 (88.2)	53 (94.6)	78 (91.8)	326 (84.5)
Anti-c	1 (20.0)	2 (11.8)	3 (5.4)	7 (8.2)	60 (15.5)
Gravidity, median [IQR]	3 [2.5-4]	2 [1.5-3]	3 [2-4]	4 [2-5]	3 [2-4]
Parity, median [IQR]	2 [1.5-3]	1 [0.5-2]	1 [1-2]	2 [1-3]	1 [1-2]
Caesarean	2 (40.0)	7 (41.2)	27 (48.2)	37 (43.5)	145 (37.9)
Gestational age at birth, median [IQR] w	33.9 [33.8-33.9]	34.7 [34.1-34.9]	35.4 [35.1-35.6]	36.4 [36.1-36.7]	37.7 [37.3-38.4]
Birthweight, median [IQR], grams	2100 [1975-2430]	2325 [2150-2700]	2628 [2362-2883]	2830 [2586-3110]	3134 [2855-3430]
Female	3 (60)	10 (58.8)	29 (51.8)	50 (58.1)	222 (57.5)
Hemoglobin level at birth, median [IQR], g/dL	15.5 [18.1-10.3]	12.7 [10.0-16.8]	12.8 [11.1-15.6]	12.9 [11.1-15.0]	15.0 [13.3-17.0]
Exchange transfusion(s)	2 (40.0)	7 (41.2)	16 (28.6)	21 (24.7)	66 (17.1)

© 2025 de Winter DP et al. *JAMA Network Open.*

eTable 7. Variance Inflation Factors of Independent Variables Included in the Analysis on Adverse Neonatal Outcome

eTable 7 shows the variance inflation factors of the independent variables that were associated with the occurrence of adverse neonatal outcome. The variance inflation factor is a measure to assess multicollinearity between independent factors. The value for variance inflation factors start at 1, that indicates no correlation between independent variables. A value between 1 and 5 shows a moderate correlation and a value greater than 5 indicates a strong correlation. Below results indicate no correlation between gestational age at birth, hemoglobin level at birth and whether an exchange transfusion was performed.

**Variable**	**Variance Inflation Factor**
Gestational age at birth (weeks)	1.084
Hemoglobin level at birth (g/dL)	1.168
Whether an exchange transfusion was performed	1.126

© 2025 de Winter DP et al. *JAMA Network Open.*

eFigure 3. Frequency of Neonatal Comorbidities and Mortality per Gestational Age at Birth

eFigure 3 displays the frequency of neonatal comorbidities and mortality per gestational age at birth.

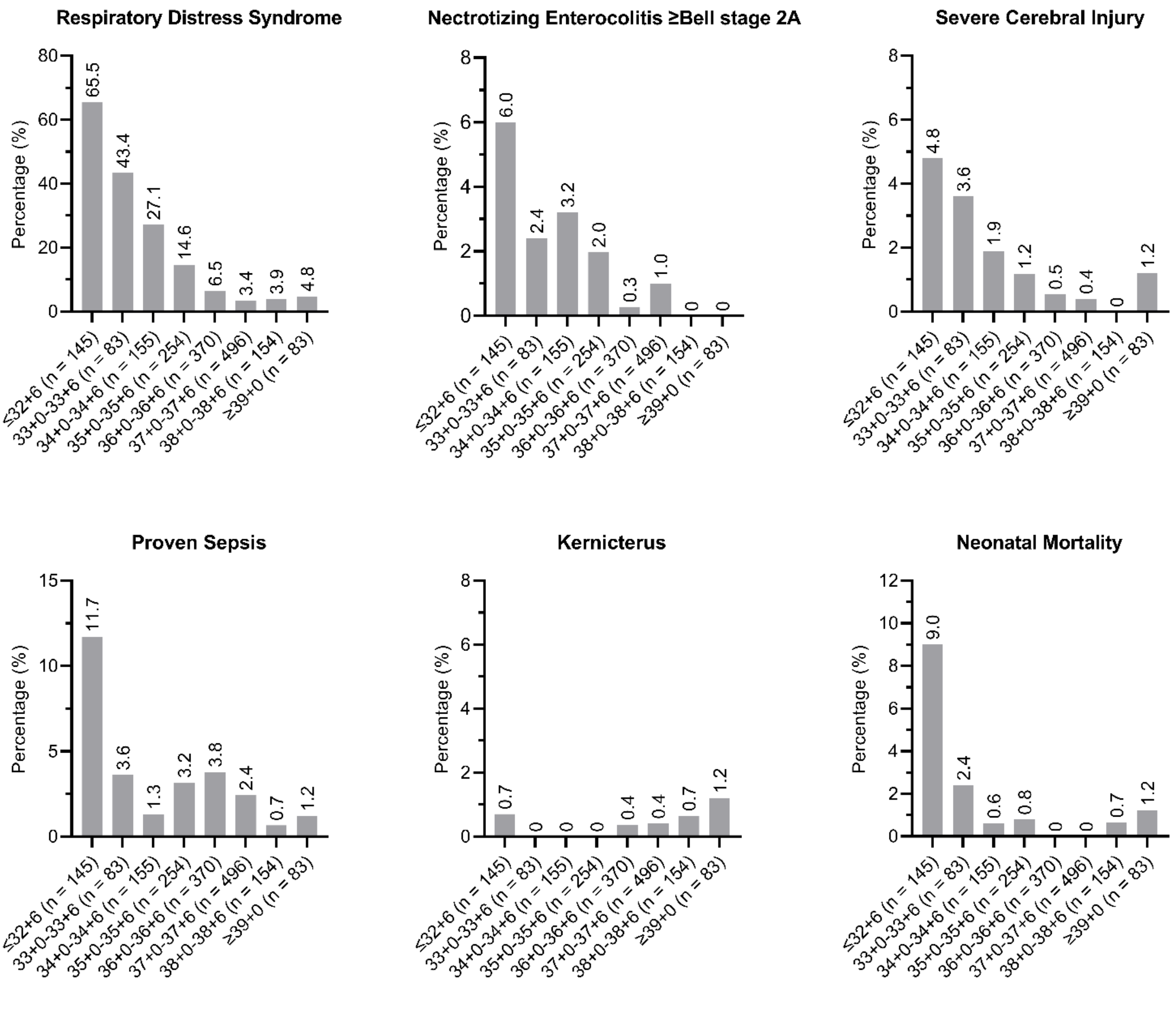

© 2025 de Winter DP et al. *JAMA Network Open.*
