# Supplementary material for: Variations and Opportunities in Postnatal Management of Hemolytic Disease of the Fetus and Newborn
Source: JAMA Netw Open. 2025 Jan 10;8(1):e2454330. doi: 10.1001/jamanetworkopen.2024.54330 (PMC11724339; doi:10.1001/jamanetworkopen.2024.54330)
Supplement: Supplement 2. — Nonauthor Collaborators. Worldwide Collaboration for Hemolytic Disease of the Fetus and Newborn (DIONYSUS) Investigators [file jamanetwopen-e2454330-s002.pdf]

Supplemental Online Content: Nonauthor Collaborators
*First name, last name, and suffix (if applicable) are required and will appear in PubMed.

*Group Name(s): Worldwide Collaboration for Hemolytic Disease of the Fetus and Newborn (DIONYSUS) Investigators							
***First Name and Middle Initial(s)**	***Last Name**	***Suffix (eg, Jr, III)**	Academic Degrees	Institution	Location (city, state/province, country)	Role or Contribution, eg, chair, principal investigator	Group (if more than 1 Group listed in the byline) and/or Subgroup (eg, Steering Committee)
Iris	Hellsing		MD	Karolinska Institutet	Stockholm, Sweden	Investigator	
Karin	Sundberg		MD, PhD	Copenhagen University Hospital, Rigshospitalet	Copenhagen, Denmark	Investigator	
Frederik B.	Clausen		PhD	Copenhagen University Hospital, Rigshospitalet	Copenhagen, Denmark	Investigator	
Eugenia	Antolin		MD, PhD	La Paz University Hospital	Madrid, Spain	Investigator	
Nieves	Mendez		MD	La Paz University Hospital	Madrid, Spain	Investigator	
Baptiste	Teillet		MD	Université de Lille, CHU Lille	Lille, France	Investigator	
Thameur	Rakza		MD	Université de Lille, CHU Lille	Lille, France	Investigator	
Erika	Hrastar		MD	University Medical Centre Ljubljana	Ljubljana, Slovenia	Investigator	
Mihael	Rus		MD	University Medical Centre Ljubljana	Ljubljana, Slovenia	Investigator	
Stefan	Verlohren		MD, PhD	Charité - Universitätsmedizin Berlin	Berlin, Germany	Investigator	
Beate	Mayer		MD, PhD	Charité - Universitätsmedizin Berlin	Berlin, Germany	Investigator	
Kerry	Rademan		MBChB	Tygerberg Academic Hospital	Stellenbosch, South Africa	Investigator	
Aline	Wolter		MD	Justus-Liebig-University	Gießen, Germany	Investigator	
Ivonne	Bedei		MD	Justus-Liebig-University	Gießen, Germany	Investigator	
Roland	Axt-Fliedner		MD, PhD	Justus-Liebig-University	Gießen, Germany	Investigator	
Yoav	Yinon		MD	Sheba Medical Centre	Tel Aviv, Israel	Investigator	
Tzipora	Strauss		MD	Sheba Medical Centre	Tel Aviv, Israel	Investigator	
Raigam Jafet	Martine-Portilla		MD	National Institute of Perinatology	Mexico City, Mexico	Investigator	
Jose A.	Montoya-Martinez		MD	National Institute of Perinatology	Mexico City, Mexico	Investigator	
Johannes	Keunen		MD, PhD	Mount Sinai Hospital	Toronto, Canada	Investigator	
Greg	Ryan		MB	Mount Sinai Hospital	Toronto, Canada	Investigator	
Francesca	Castoldi		MD, PhD	Buzzi Children’s Hospital	Milan, Italy	Investigator	
Chiara	Nava		MD	Buzzi Children’s Hospital	Milan, Italy	Investigator	
Philipp	Klaritsch		MD, PhD	Medical University of Graz	Graz, Austria	Investigator	
Mabel Laura	Cabrera		MD	Hospital de La Mujer	La Paz, Bolivia	Investigator	
Alexandra	Matias		MD, PhD	Unidade Local de Saúde de São João	Porto, Portugal	Investigator	
Fangfang	Tao		MD	Shanghai First Maternity and Infant Hospital	Shanghai, China	Investigator	
Jiangqin	Liu		MD	Shanghai First Maternity and Infant Hospital	Shanghai, China	Investigator	

Supplemental Online Content: Nonauthor Collaborators
*First name, last name, and suffix (if applicable) are required and will appear in PubMed.

***First Name and Middle Initial(s)**	***Last Name**	***Suffix (eg, Jr, III)**	Academic Degrees	Institution	Location (city, state/province, country)	Role or Contribution, eg, chair, principal investigator	Group (if more than 1 Group listed in the byline) and/or Subgroup (eg, Steering Committee)
Leandro Daniel	Burgos Pratx		MD	Instituto Universitario Hospital Italiano de Buenos Aires	Buenos Aires, Argentina	Investigator	
Mark	Kilby		DSc, MD	Birmingham Women's and Children's NHS Foundation Trust	Birmingham, UK	Investigator	
Rob	Negrine		MD	Birmingham Women's and Children's NHS Foundation Trust	Birmingham, UK	Investigator	
