# Supplementary material for: Variations and Opportunities in Postnatal Management of Hemolytic Disease of the Fetus and Newborn
Source: JAMA Netw Open. 2025 Jan 10;8(1):e2454330. doi: 10.1001/jamanetworkopen.2024.54330 (PMC11724339; doi:10.1001/jamanetworkopen.2024.54330)
Supplement: Supplement 3. — Data Sharing Statement [file jamanetwopen-e2454330-s003.pdf]

Data Sharing Statement

de Winter. Variations and Opportunities in Postnatal Management of Hemolytic Disease of the Fetus and Newborn. *JAMA Netw Open*. Published January 10, 2025. doi:10.1001/jamanetworkopen.2024.54330

Data
Data available: No

Additional Information
Explanation for why data not available: The data that support this study cannot be shared openly due to reasons of sensitivity and to protect the participants privacy. Data are located in a controlled access data storage at Leiden University Medical Center.
